# Three-dimensional electron microscopy reveals the evolution of glomerular barrier injury

**DOI:** 10.1038/srep35068

**Published:** 2016-10-11

**Authors:** Michael J. Randles, Sophie Collinson, Tobias Starborg, Aleksandr Mironov, Mira Krendel, Eva Königshausen, Lorenz Sellin, Ian S. D. Roberts, Karl E. Kadler, Jeffrey H. Miner, Rachel Lennon

**Affiliations:** 1 Wellcome Trust Centre for Cell-Matrix Research, Faculty of Life Sciences, University of Manchester, UK; 2 Institute of Human Development, Faculty of Medical & Human Sciences, University of Manchester, UK; 3 Electron Microscopy Core Facility, Faculty of Life Sciences, University of Manchester, UK; 4 Department of Cell and Developmental Biology, SUNY Upstate Medical University, Syracuse, New York, USA; 5 Department of Nephrology, Medical School, Heinrich Heine University, Düsseldorf, Germany; 6 Department of Cellular Pathology, John Radcliffe Hospital, Oxford, UK; 7 Division of Nephrology, Washington University School of Medicine, St Louis, Missouri, USA

## Abstract

Glomeruli are highly sophisticated filters and glomerular disease is the leading cause of kidney failure. Morphological change in glomerular podocytes and the underlying basement membrane are frequently observed in disease, irrespective of the underlying molecular etiology. Standard electron microscopy techniques have enabled the identification and classification of glomerular diseases based on two-dimensional information, however complex three-dimensional ultrastructural relationships between cells and their extracellular matrix cannot be easily resolved with this approach. We employed serial block face-scanning electron microscopy to investigate Alport syndrome, the commonest monogenic glomerular disease, and compared findings to other genetic mouse models of glomerular disease (*Myo1e−*/*−, Ptpro−*/*−*). These analyses revealed the evolution of basement membrane and cellular defects through the progression of glomerular injury. Specifically we identified sub-podocyte expansions of the basement membrane with both cellular and matrix gene defects and found a corresponding reduction in podocyte foot process number. Furthermore, we discovered novel podocyte protrusions invading into the glomerular basement membrane in disease and these occurred frequently in expanded regions of basement membrane. These findings provide new insights into mechanisms of glomerular barrier dysfunction and suggest that common cell-matrix-adhesion pathways are involved in the progression of disease regardless of the primary insult.

Glomerular disease is characterised by persistent proteinuria, with or without haematuria, and a progressive decline in renal function. Currently, no curative therapy exists and patients are reliant upon non-specific treatments. Glomerular dysfunction can result from genetic defects, inflammatory insults or can be secondary to systemic disease such as diabetes mellitus. Alport syndrome is the commonest monogenic glomerular disease and is characterised by progressive glomerular dysfunction, sensorineural deafness and ocular abnormalities^1^. Patients with Alport syndrome are treated with renin-angiotensin-aldosterone system (RAAS) inhibitors and this therapy has been shown to prolong renal function by over 15 years^2^. However, many patients ultimately progress to end stage renal disease and more effective therapies are urgently needed.

The molecular origin of Alport Syndrome was first described in 1990 when mutations in *COL4A5* were reported^3^. *COL4A5* is expressed on the X-chromosome and therefore hemizygous males are more severely affected than heterozygous females^3^,^4^. Subsequently, mutations in *COL4A3* and *COL4A4* were discovered and in this scenario two mutated alleles cause autosomal recessive disease^5^,^6^,^7^. Within the kidney, glomerular podocytes are the only cells known to express *COL4A3, COL4A4* and *COL4A5*^8^, and these genes are required for the assembly of the type IV collagen α3α4α5 network in the glomerular basement membrane (GBM). The GBM is a complex niche of extracellular matrix (ECM) proteins that is essential for normal glomerular function^9^. In the adult glomerulus the GBM is composed of predominately type IV collagen α3α4α5, laminin α5β2γ1 and up to 70 additional structural and regulatory components^10^. Evidence suggests that type IV collagen α3α4α5 is important for the lifelong structural integrity of the GBM^11^, as the type IV collagen α1α1α2 network, produced by both podocytes and glomerular endothelial cells^12^, is unable to compensate for the absence of the α3α4α5 network in Alport Syndrome^13^,^14^.

Renal biopsy is a valuable diagnostic tool for defining the sub-type of glomerular disease; indeed a diagnostic marker for Alport Syndrome is the pathological basket weave appearance of the GBM, although genetic testing is now superseding biopsy^15^. Other histopathological features of glomerular disease include altered GBM thickness, loss of unique podocyte structures called foot processes (FPs) and a reduction in podocyte number^16^. Interestingly, these features are reported across the spectrum of glomerular disease regardless of the underlying molecular aetiology. Yet the mechanisms underpinning these morphological changes are poorly understood. In particular we have not defined the ultrastructural relationship between podocytes and the GBM during the progression of glomerular disease, however this knowledge could provide insight into disease mechanisms.

To address this knowledge gap we employed serial block face-scanning electron microscopy (SBF-SEM) to examine the relationships of cells and ECM in three-dimensions (3D). This approach was recently used to investigate cell-ECM interactions^17^,^18^,^19^,^20^ and the complexities of normal podocyte structure^21^. However, until now, the approach has not been used to investigate changes in podocytes and the GBM during disease progression. We hypothesized that SBF-SEM over a time course would enable the construction of detailed 3D models of the glomerulus in health and disease and would reveal novel pathological features.

## Results

### 3D modelling of glomerular structure in Alport nephropathy

**Figure 1:**
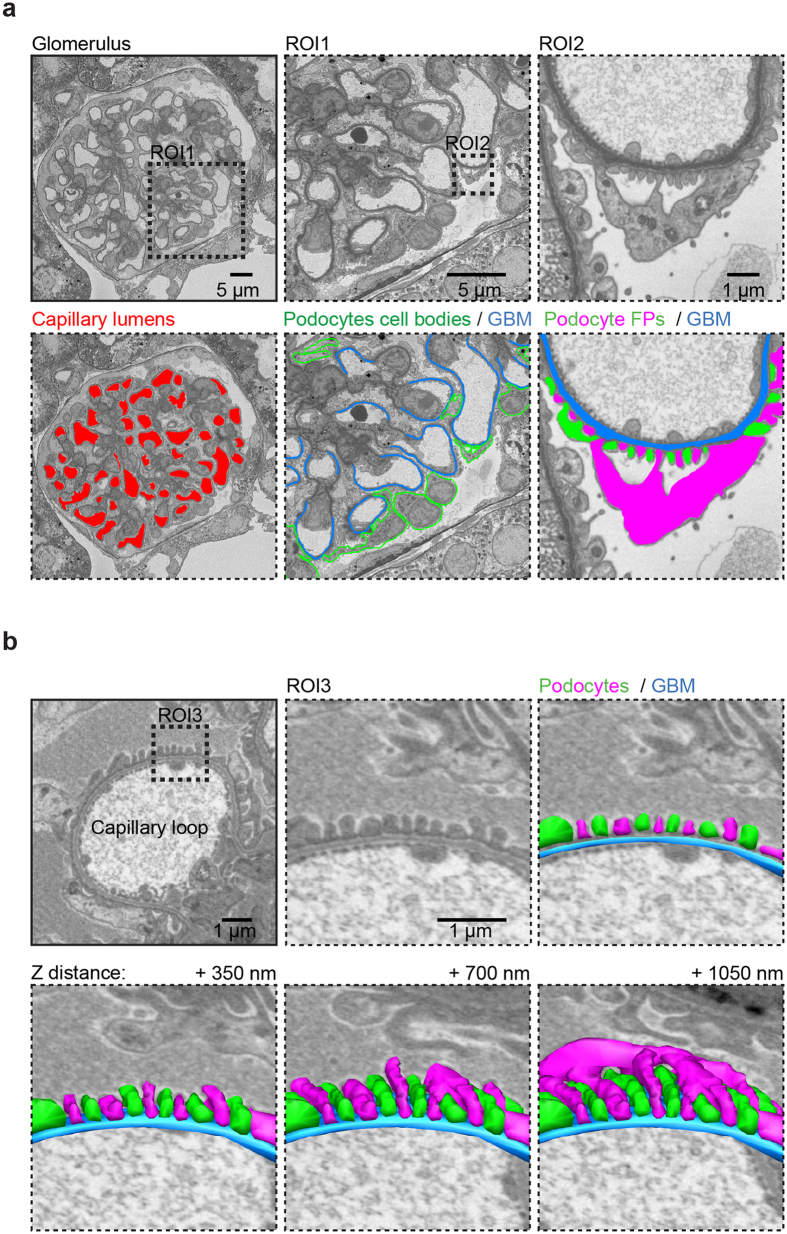
The 3D morphology of podocyte foot processes and the GBM. 3D reconstructions from SBF-SEM images of wild type glomerulus from an 18-week C57Bl/6 mouse. (**a**) A single slice from an SBF-SEM stack showing a glomerulus in cross section. Glomerular capillary lumens are highlighted in red, the GBM in blue and podocyte cell bodies in green. Regions of interest (ROIs) show that podocyte FPs from two adjacent podocytes (one green the other magenta) can clearly be discerned using this technique. (**b**) Panel displays a transverse view of reconstructed podocyte FPs and GBM. Removing slices (increasing Z distance) reveals the 3-dimensional organization of the foot processes. Serial block face-scanning electron microscopy, SBF-SEM; glomerular basement membrane, GBM; foot processes, FPs.

**Figure 2:**
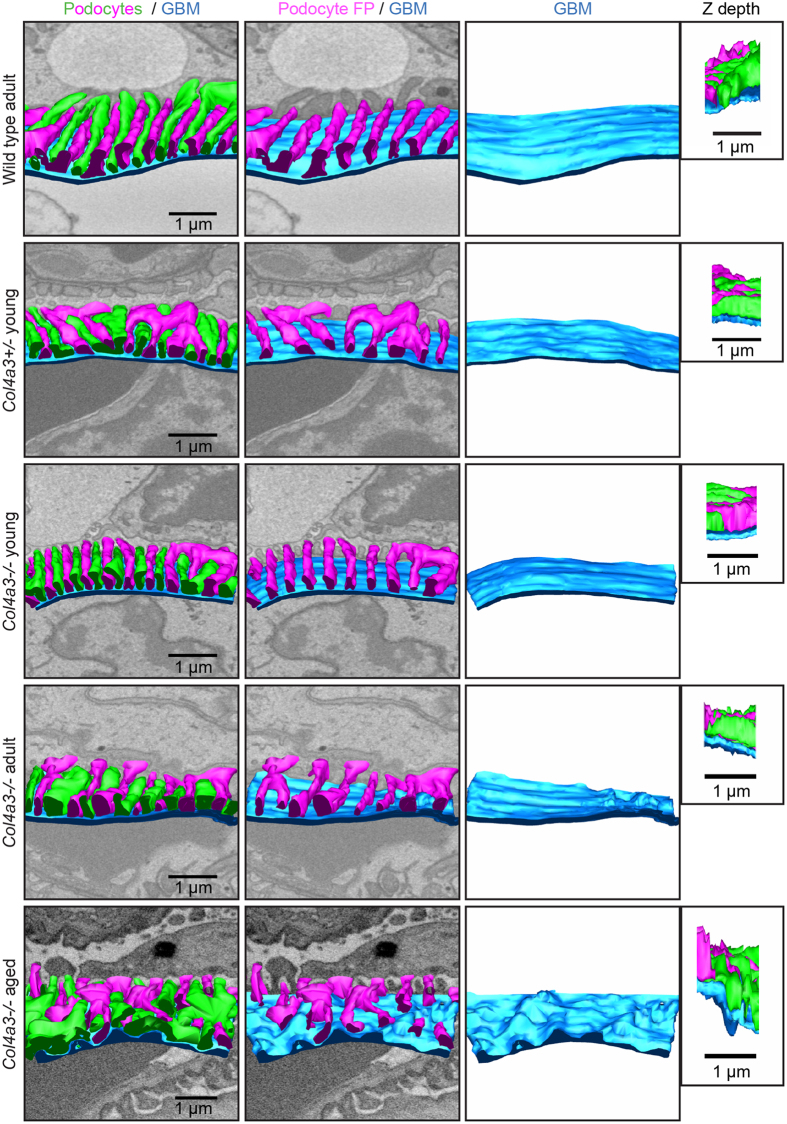
Normal podocyte FP and GBM structure is lost with age in Alport mice. Transverse views of reconstructed GBMs (blue) and podocyte FPs (green and magenta) from wild type, *Col4a3−*/*−* mice and *Col4a3−*/*−* mice. Many regions in young adult (young) and older adult (adult) *Col4a3−*/*−* mice display organized podocyte FPs and uniform GBM of thickness comparable to wild type adult animals. GBM, glomerular basement membrane; FPs, foot processes.

**Figure 3:**
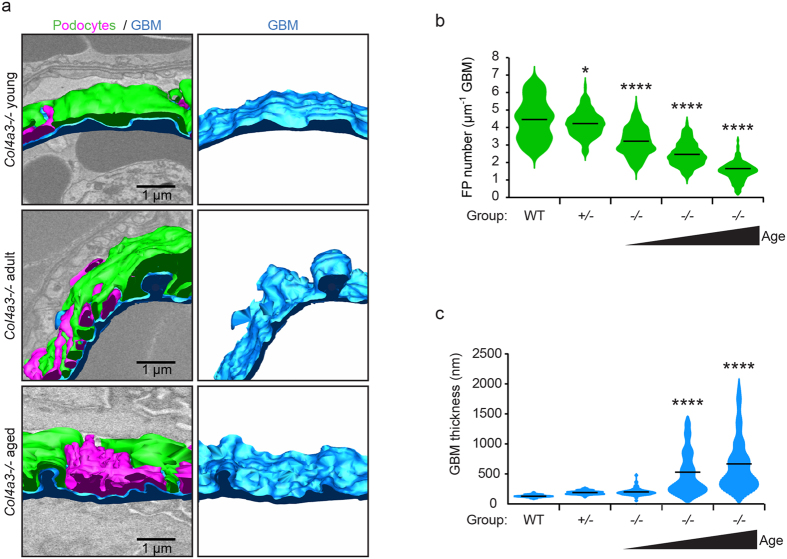
3D analysis of glomerular structure in aging *Col4a3−*/*−* mice. (**a**) Transverse view of reconstructed GBMs (blue) and podocyte FPs (green and magenta) reveal focal areas of podocyte FP effacement that coincide with thickened irregular regions of GBM. (**b**) Quantification of podocyte FP density. The violin plots demonstrate the distributions for the number of FPs per length of GBM along the y-axis, the black lines represent the mean number of FPs per length of GBM in each group. (**C**) Quantification of GBM thickness. The violin plots describe the distributions for the thickness of GBM along the y-axis, the black lines represent the mean thickness of the GBM in each group. GBM, glomerular basement membrane; FPs, foot processes; *P < 0.05; ****P < 0.0001.

Over a 28-week time course we investigated *Col4a3−*/*−* male mice on a C57BL/6J genetic background. We selected this background, which has a slow progression of glomerular disease, to allow a greater window of observation. We analyzed glomeruli using SBF-SEM and created 3D glomerular reconstructions (Supplementary Movie S1). The glomerulus is composed of interwoven capillary loops forming a complex pattern of lumens when viewed in 3D. The localization of the GBM, podocyte cell bodies and foot processes (FPs) can be easily discerned using SBF-SEM (Fig. 1a). From wild type 16–18 week (older adult) mouse glomeruli we generated models of podocytes and GBM and these revealed an organized glomerular structure (Fig. 1a,b). We compared these models with glomeruli from 6-week (young adult) *Col4a3*+/*−* and *Col4a3−*/*−* animals and found that the majority of podocytes had regular FPs (Fig. 2). This finding was also observed in older adult *Col4a3−*/*−* mice although some FPs were flattened or effaced (Fig. 2). In contrast, 28-week (aged) *Col4a3−*/*−* mice had striking global loss of podocyte FP organization, GBM thickening and thinning (Fig. 2). Further analysis revealed focal regions of reduced podocyte FP density in all groups of *Col4a3−*/*−* mice, even in young adult mice, when compared with age matched wild type and *Col4a3*+/*−* mice (Fig. 3a,b). These areas were often concomitant with thickened, non-uniform sheets of GBM (Fig. 3a,c and Supplementary Movies S2 and S3). Moreover, the density of podocyte FPs decreased with age in *Col4a3−*/*−* mice (Fig. 3b) and correlated with increased thickness and variation in thickness of the GBM (Fig. 3c).

### Podocytes invade into the GBM in Alport nephropathy

**Figure 4:**
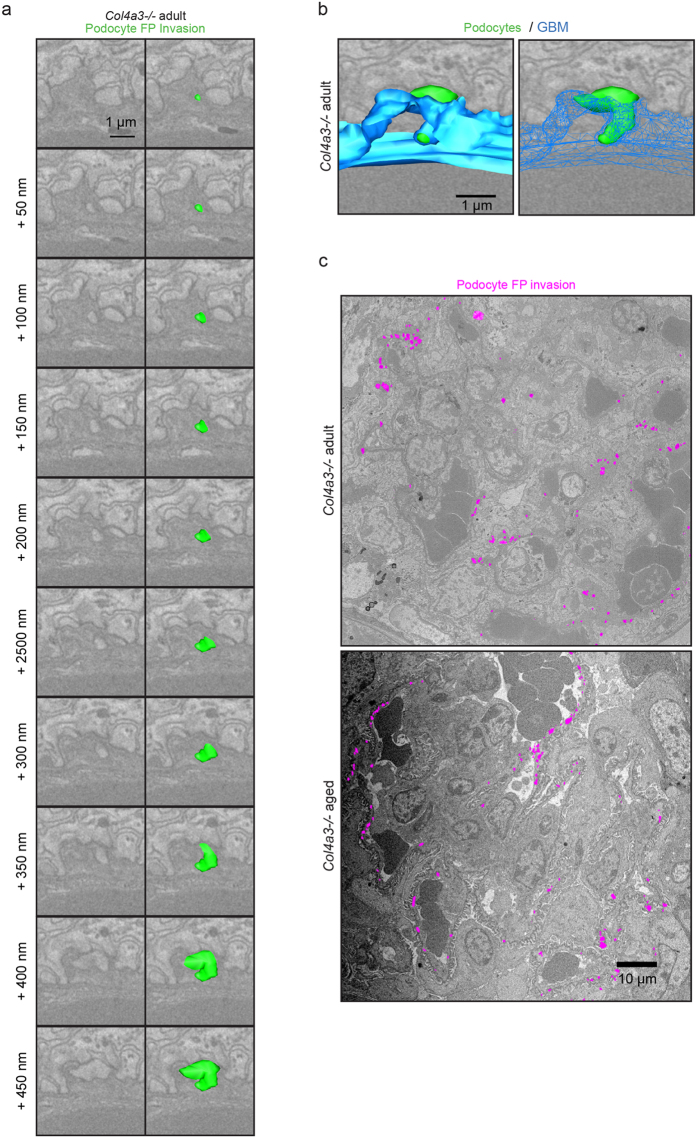
Identification of podocyte FPs invading the GBM in Alport syndrome. (**a**) Tracking of cellular material embedded within the GBM through 50 nm sections revealed that this material originates from podocyte FPs. (**b**) Tilted view of the model generated in A. showing the interaction of the invading FP with the BM. (**c**) Distribution of GBM invasions of podocyte origin (magenta) recorded in 10,000 μm^3^ glomerular volume. Podocyte FP invasions were not present in wild type adult or *Col4a3*+/*−* mice, they were rarely observed in young *Col4a3−/*mice, but were abundant in adult and aged *Col4a3−*/*−* mice. GBM, glomerular basement membrane.

**Figure 5:**
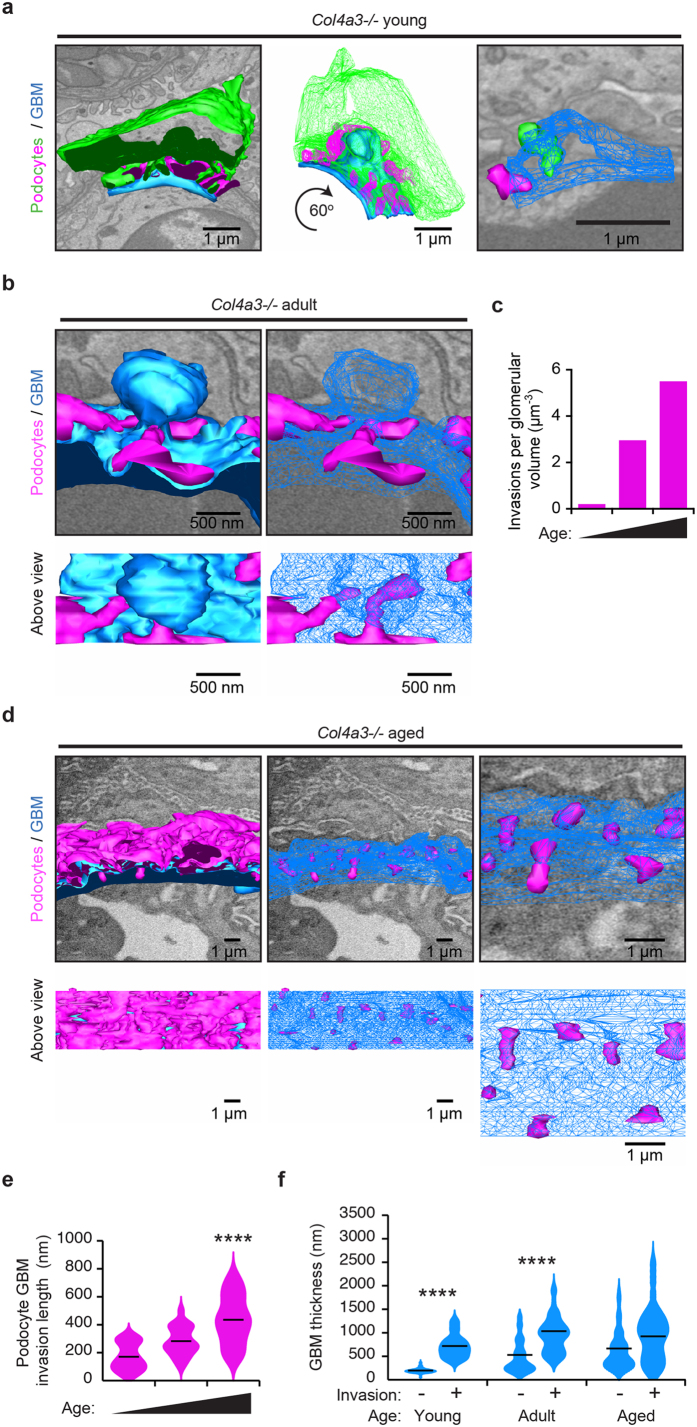
Analysis of podocyte FP invasions in Alport mice. (**a**) Reconstructed podocyte GBM interface in a young adult (6 week) *Col4a3−*/*−* mouse. Podocyte cell body (green) and FPs from a neighbouring podocyte (magenta) are shown. A rare example of two short podocyte FP invasions inside the GBM (blue) is shown in young *Col4a3−*/*−* mice (right hand panel). The GBM in this region in thickened relative to the rest of the GBM. (**b**) SBF-SEM reconstruction reveals two FPs invading an abnormally thickened area of GBM in an adult (16 week) *Col4a3−*/*−* mouse. (**c**) The number of FP invasions increase with age in *Col4a3−*/*−* mice. (**d**) FP invasions are frequent in aged (28 week) *Col4a3−*/*−* mice. The GBM is irregular in thickness, both thinned and thickened compared to young GBM. (**e**) Quantification of podocyte FP invasions. The violin plots describe the distributions for the length of podocyte invasion into the GBM along the y-axis, the black lines represent the mean length of podocyte GBM invasions in each group. (**f**) Quantification of GBM thickness at sites of podocyte invasion. The violin plots describe the distributions for the thickness of GBM at sites of podocyte invasion and sites where there is no podocyte invasion. The black lines represent the mean GBM thickness of each group. GBM, glomerular basement membrane; FPs, foot processes; *P < 0.05; ****P < 0.0001.

The appearance of cellular material within the GBM has been previously described. Erythrocytes have occasionally been captured traversing the GBM in thin basement membrane nephropathy^22^. Podocyte infolding in the GBM has also been described in a range of glomerular pathologies^23^ and more recent investigation of *Col4a3−*/*−* mice, suggested that cellular interposition in the GBM is of mesangial cell origin^24^. Mesangial cells are located between adjacent capillary loops and have contact with the GBM at the bases of the capillary loops (Supplementary Fig. S1a). In contrast, podocytes directly adhere to the GBM of the capillary walls (Fig. 1a,b). Using SBF-SEM we were able to identify cellular invasions within the GBM that connected to podocyte FPs (Fig. 4a,b and Supplementary Movies S4 and S5). These invasions were rarely observed in young adult *Col4a3−*/*−* mice (Fig. 5a) and were never observed in *Col4a3*+/*−* or wild type mice. In contrast, older adult and aged *Col4a3−*/*−* mice had frequent podocyte invasions (Figs 4c and 5b–d). The lengths of podocyte invasions were variable, but the mean length increased with age (Fig. 5e). Moreover, they occurred more frequently in thickened and abnormal areas of GBM in *Col4a3−*/*−* mice (Figs 4c and 5f). In aged *Col4a3−*/*−* mice the entire GBM had irregular thickness and all regions of the GBM contained podocyte invasions (Fig. 5d,f). Since it is known that the genetic background of Alport mice has an effect on the rate of disease progression^25^, we also investigated *Col4a3−*/*−* mice on a 129S1/Svlmj background with SBF-SEM. Here, similarly, we found evidence of podocytes invading into the GBM (Supplementary Fig. S2a–e).

### Podocyte-GBM invasion is a common feature in glomerular disease

**Figure 6:**
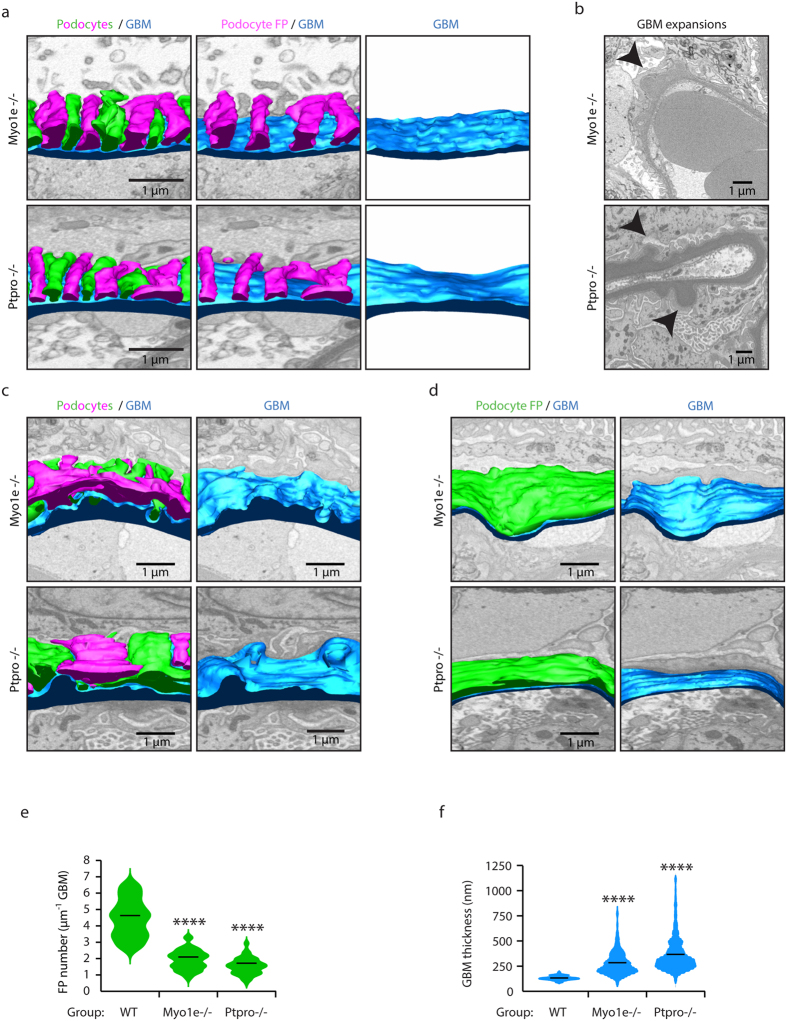
3D analysis of *Myo1e−*/*−* and *Ptpro−*/*−* mouse glomerular structure. (**a**) Transverse view of reconstructed GBMs (blue) and podocyte FPs (green and magenta) reveal broadened and flattened FPs in *Myo1e−*/*−* and *Ptpro−*/*−* mice. (**b**) Large expansions of GBM occur in *Myo1e−*/*−* and *Ptpro−*/*−* mice. (**c**) Focal areas of podocyte FP effacement coincide with thickened and irregular regions of GBM in *Myo1e−*/*−* and *Ptpro−*/*−* mice. (**d**) In many regions there is complete effacement of podocyte FPs in in *Myo1e−*/*−* and *Ptpro−*/*−* mice. (**e**) Quantification of podocyte FP density. The violin plots describe the distributions for the number of FPs per length of GBM along the y-axis, the black lines represent the mean number of FPs per length of GBM in each group. (**f**) Quantification of GBM thickness. The violin plots describe the distributions for the thickness of GBM along the y-axis, the black lines represent the mean thickness of the GBM in each group. GBM, glomerular basement membrane; FPs, foot processes; ****P < 0.0001.

**Figure 7:**
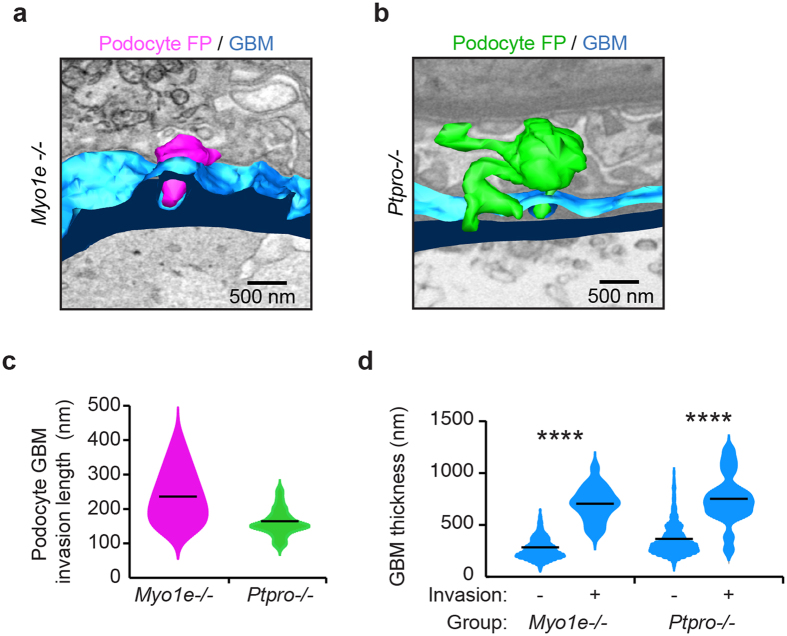
Podocyte-GBM invasion is a common feature in glomerular disease. (**a**) Reconstructed podocyte GBM invasion in *Myo1e−*/*−* mouse. Podocyte invasion (magenta) and GBM (blue) are shown. (**b**) Reconstructed podocyte GBM invasion in *Ptpro−*/*−* mouse. Podocyte invasion (green) and GBM (blue) are shown. (**c**) Quantification of podocyte FP invasions. The violin plots describe the distributions for the length of podocyte invasion into the GBM along the y-axis, the black lines represent the mean length of podocyte GBM invasions in each group. (**d**) Quantification of GBM thickness at sites of podocyte invasion. The violin plots describe the distributions for the thickness of GBM at sites of podocyte invasion and sites where there is no podocyte invasion. The black lines represent the mean GBM thickness of each group. GBM, glomerular basement membrane; FPs, foot processes; ****P < 0.0001.

To investigate whether podocyte invasion into the GBM is a unique feature of Alport Syndrome, or shared with other genetic glomerular diseases, we investigated two additional mouse models of human disease. *MYO1E* mutations in humans cause steroid resistant focal segmental glomerulosclerosis (FSGS)^26^. *MYO1E* encodes myosin1e, a class I myosin which is expressed by mouse and human podocytes^26^,^27^. *Myo1e−*/*−* mice develop FSGS and renal failure associated with morphological abnormalities in both podocytes and the GBM; we therefore investigated adult *Myo1e−*/*−* mouse glomeruli with SBF-SEM. Our analysis revealed that podocyte FPs were flattened, broadened and in many regions completely effaced (Fig. 6a–c). There were expanded areas of GBM that coincided with loss of podocyte FP morphology (Fig. 6d). Overall, there was a loss of podocyte FP density and a thickening of the GBM (Fig. 6f,g). Along with these classical pathological changes in podocyte and GBM ultrastructure, we also discovered prevalent podocyte FP invasion into the GBM (Fig. 7a, Supplementary Fig. S3a and Supplementary Movie S6).

Next we investigated a third mouse model of human glomerular disease. Glomerular epithelial protein 1 (GLEPP1) is a receptor tyrosine phosphatase expressed by podocytes. Mutations in *PTPRO* the gene that encodes GLEPP1 protein causes childhood-onset nephrotic syndrome in humans^28^. *Ptpro−*/*−* mice have impaired renal function and SEM has demonstrated that these mice have shortened podocyte FPs^29^. We investigated adult *Ptpro−*/*−* mice with SBF-SEM and found dramatic GBM expansions, overall GBM thickening and focal areas of reduced podocyte FP density (Fig. 6a–d,f,g). Furthermore we identified podocyte invasions into the GBM similar to those observed in *Col4a3−*/*−* mice and *Myo1e−*/*−* mice (Supplementary Movie S7). In contrast to *Col4a3−*/*−* and *Myo1e* mice, podocyte invasions into the GBM in *Ptpro−*/*−* mice were more slender and short (Fig. 7b and Supplementary Fig. S3b). In addition, podocyte invasions in *Ptpro−*/*−* mice were less variable in length than those in adult and aged *Col4a3−*/*−* mice (Fig. 7c). Similar to *Col4a3−*/*−* mice, podocyte invasions were associated with thickened regions of GBM in both *Myo1e−*/*−* and *Ptpro−*/*−* mice (Fig. 7d).

### Mesangial cells invade the GBM at the mesangial aspect

Using 3D modelling of glomerular structure we also analysed the mesangial cells since they have been reported to invade the GBM in Alport syndrome^24^. Indeed, analysing SBF-SEM from Alport mice we identified mesangial invasions of varying length inside the GBM along the mesangial aspect of the glomerular capillary loop (Supplementary Fig. S1b and Supplementary Movies S9 and S10). These mesangial processes were distinct from podocyte invasions; they were only observed close to the mesangial angle and not in the peripheral capillary loops. We also detected mesangial invasions in both *Myo1e−*/*−* and *Ptpro−*/*−* mice (Supplementary Fig. S1c,d and Supplementary Movies S10 and S11). Mesangial invasions were rarer than podocyte invasions and in contrast to podocyte invasions, mesangial invasions were also present in wild type animals (Supplementary Movie S8). However, mesangial invasions were longer in mice with *Col4a3, Myo1e* and *Ptpro* mutations compared with wild type mice (Fig. S1E, Movie S9–11).

### GBM defects resembling podocyte invasion in human Alport syndrome

**Figure 8:**
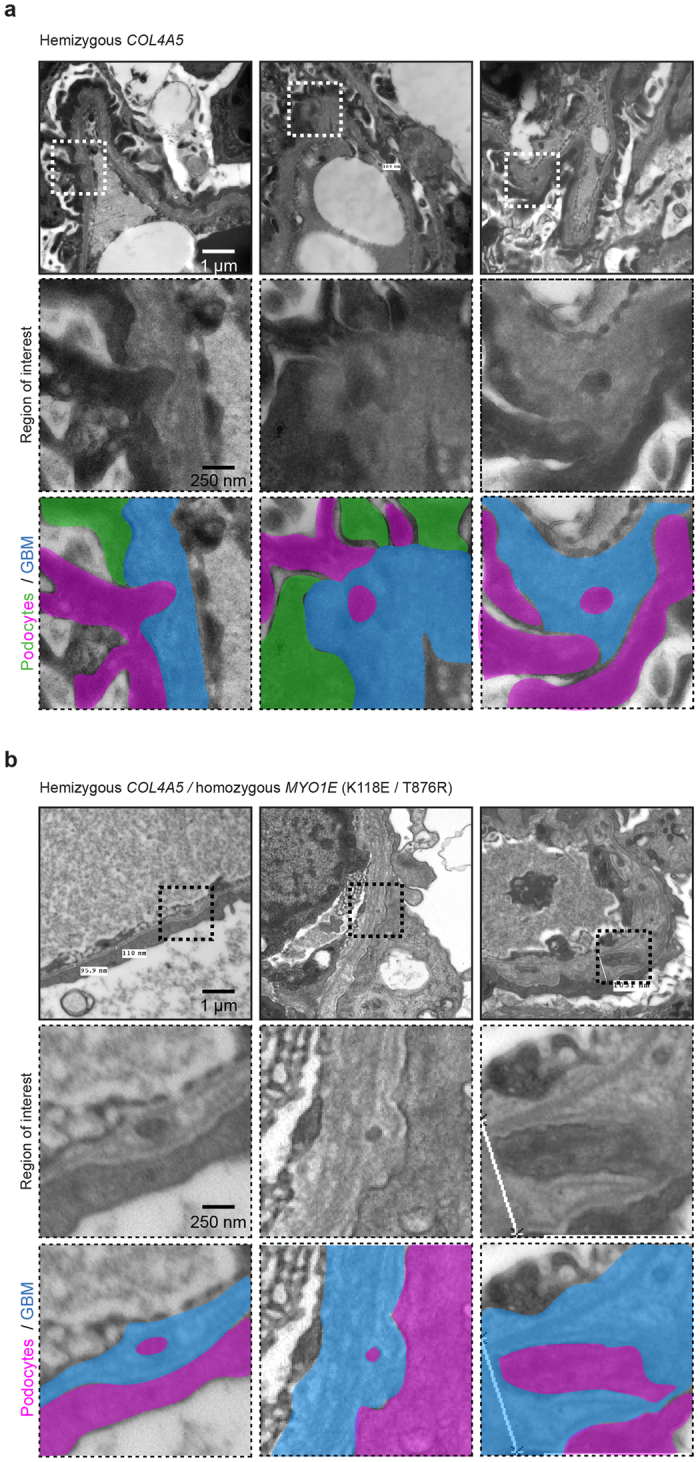
Evidence for podocyte invasion in patients with Alport syndrome. Transmission electron microscopy of an Alport patient with mutant *COL4A5* alleles c.2858G > T; p.(Gly953Val) and c.3097G > C; p.(Gly1033 Arg) (**a**) and a sibling who in addition to carrying *COL4A5* variants had mutations in *MYO1E:* c.352A > G; p.(Lys118Glu) and c.2627C > G; p.(Thr876Arg) (**b**) These TEMs reveal features that resemble podocyte invasions identified in mouse models of glomerular disease.

We interrogated TEM sections from the renal biopsies of two siblings that we have previously reported^30^. The proband was investigated for persistent microscopic haematuria and mild proteinuria and he had a renal biopsy at 3 years of age. This demonstrated abnormal GBM thickness, which prompted *COL4* genetic screening. He was found to be hemizygous for two *COL4A5* variants: c.2858G > T; p.(Gly953Val) and c.3097G > C; p.(Gly1033 Arg). His younger brother presented at the age of 6 months with steroid resistant nephrotic syndrome and was also hemizygous for the same *COL4A5* variants. In addition he carried two homozygous missense variants in *MYO1E*: c.352A > G; p.(Lys118Glu) and c.2627C > G; p.(Thr876Arg). In both renal biopsies there was evidence of electron lucent regions within the GBM (Fig. 8). In addition, there was evidence of cellular material within the GBM and these resembled the podocyte invasions that we observed in *Col4a3−*/*−, Myo1e−*/*−* and *Ptpro−*/*−* mice. Future SBF-SEM studies of human biopsies are needed to confirm that the cellular material is indeed podocyte protrusions as we have shown in the mouse.

## Discussion

In this study we used SBF-SEM to analyze spatial relationships between cellular and extracellular glomerular components in health and disease. We identified podocyte invasions into the GBM as a novel morphological feature of glomerular disease. Irregular expansions of the urinary surface of the GBM were also a dominant feature in all disease models and these irregular expansions were more likely to harbor podocyte invasions. Podocyte invasions occurred in Alport (*Col4a3−*/*−), Myo1e−*/*−* and *Ptpro−*/*−* mice and lucent regions of GBM, resembling sites of podocyte invasion, were seen in patients with Alport syndrome. Finally, we demonstrate that GBM thickness and irregularity markedly increases and podocyte FP density decreases with glomerular disease progression.

The GBM is a critical component of the glomerular filtration barrier being exquisitely water permeable yet prohibiting the passage of macromolecules. Super resolution microscopy was recently used to describe the precise molecular topology of the GBM^31^. In this nanoscale investigation of the GBM, type IV collagen was shown to be restricted to the centre of the GBM. This localization indicates that the collagen IV network is too distant from podocytes to interact with integrins at the cell surface and therefore initiate outside-in signaling. This paper also demonstrated that type IV collagen α1α1α2 mislocalizes during Alport syndrome and is found adjacent to podocytes. Type IV collagen α1α1α2 that is proximal to podocytes could conceivably perturb cell-ECM interactions or alter the organization of other components within the GBM. In our investigation, we identified novel podocyte protrusions into the GBM and one explanation for podocyte invasion is that increased GBM degradation and subsequent podocyte migration occur in order to improve podocyte anchorage and to prevent detachment. A second, attractive explanation is that podocytes use invasions to remodel GBM damage that is caused by altered filtration forces. Potentially, podocyte invasions are extensions of plasma membrane used to remodel ECM, similar to fibropositors that regulate the direction of collagen fibril assembly during tendon repair^24^.

Our investigation of human GBM was limited to TEM sections since archival EM blocks from human biopsies are not routinely stained with heavy metals prior to embedding. For SBF-SEM staining is required prior to embedding since sectioning is automated within the microscope. In the human TEM we observed lucent regions in the GBM, resembling the regions in the mouse GBM that were tracked to podocyte protrusions and we therefore propose that the same protrusions occur in human glomerular disease. These observations are likely to overlap with the previously reported podocyte infolding in the GBM, seen across a range of glomerular pathologies^23^.

Defining the molecular composition and nature of podocyte invasions into the GBM would determine whether these could be therapeutically blocked or activated in order to protect glomerular function. There are a number of candidate pathways that could drive podocyte invasion into the GBM. One such pathway is integrin mediated adhesion signalling. Focal adhesion kinase (FAK), a key integrin signalling protein, has been shown to be activated in Alport mouse podocytes by ectopic laminin α2^32^. This same study revealed that FAK induces matrix metalloproteinase (MMP) release from podocytes. This proposes an explanation for podocyte protrusion into the GBM following podocyte-mediated ECM degradation. Indeed TEM imaging provides evidence of an electro-lucent halo around FP invasions (Figure S4), which could be the result of active ECM degradation by podocytes.

In conclusion, SBF-SEM has provided enhanced visualization of the glomerulus and this has led to the identification of previously unseen pathological features of glomerular disease. 3D ultrastructural imaging revealed expected GBM abnormalities in addition to podocyte protrusions, resembling invadosomes, in the GBM. This novel finding provides new insight into disease mechanisms and suggests that a common matrix-adhesion pathway is activated regardless of the primary molecular insult. What remains unknown is whether podocytes actively migrate into the GBM or if these protrusions occur passively. Further study is necessary to understand how and why podocytes protrude into the GBM. Answers to these questions could lead to the development of targeted therapies that would offer new therapeutic avenues for glomerular disease.

## Methods

### Mouse models

All animal experiments were performed in accordance with the relevant institutional approvals. The *Col4a3−*/*−* mice have been reported previously^33^ and experiments on these mice conformed to the National Institutes of Health Guide for the Care and Use of Laboratory Animals and were approved by the Washington University Animal Studies Committee. For *Myo1e−*/*−* mice^27^ experiments were performed according to the protocol approved by the SUNY Upstate Animal Committee. *Ptpro−*/*−* mice^29^ were backcrossed onto a 129P3/J background^34^ and the experiments were approved by Heinrich Heine University, Düsseldorf. C57BL/6JOIaHsd male mice were used as WT controls. A minimum of 3 animals per group were analysed in this study.

### Serial block face-scanning electron microscopy

Kidney samples were prepared for SBF-SEM as described previously^17^. In brief, mouse kidneys were cut into 1 mm cubes and fixed *in situ* by using 2% (wt/vol) glutaraldehyde (Agar Scientific, UK) in 0.1 M cacodylate buffer (pH 7.2); stained in 1% (wt/vol) osmium tetroxide, 1.5% (wt/vol) potassium ferrocyanide in 0.1 M cacodylate buffer, followed by 1% (wt/vol) thiocarbohydrazide. After washing, more osmium was added by staining in 1% (wt/vol) osmium tetroxide, and soaked in 1% (wt/vol) uranyl acetate overnight. The final staining step incubation was performed at 60 °C with lead aspartate pH 5.5 for one hour. Samples were dehydrated in ethanol and infiltrated in TAAB 812 hard resin. Tissue was mounted onto an aluminium cryo pin (Electron Microscopy Sciences, cat. no. 70446) using cyanoacrylate glue and all block surfaces trimmed at 90° using a glass knife or diamond trimming tool (Diatome). A gold coating was applied to the block to create a conductive surface. The block was placed in the Quanta 250 FEG (FEI Company) + Gatan 3view system and a 41 μm × 41 μm field of view was chosen and imaged by using a 4096 × 4096 scan, which gave an approximate pixel size of 10 nm. The section thickness was set to 50 nm in the Z (cutting) direction.

### Transmission electron microscopy and quantification

Sample fixation and staining was performed as for SBF-SEM. Different blocks from the same sample were used for TEM and SBF-SEM. Tissue blocks were removed from their moulds and sectioned (70–80 nm thickness). Sections were examined using a FEI Tecnai 12G2 Biotwin transmission electron microscope. Magnification varied from 145X to 6800X. *n* = *6* glomeruli were imaged for each mouse model. Images were saved as original.dm3 files and.tiff files. The dm3 files were opened using Fiji/ImageJ software version 2.0.0-rc-29/1.49r. Collectively 60 randomly selected points of the GBM across 50.dm3 images were measured to assess GBM thickness. The straight-line option was used to draw across the width of the GBM at the chosen point > Analyse > Measure. To quantify podocyte FP number and FP protrusions 50 randomly selected areas of GBM 5 μm in length were measured as above and FP or protrusion number counted manually. Where protrusions were identified the thickness of the infiltrated GBM was also measured. All measurements were imported into GraphPad Prism software version 5.0 for interpretation and graphical representation.

### 3D modeling

Typically, Z volumes datasets comprised 1000 images (50 μm z depth). The IMOD beta version 4.7.11 suite of image analysis software was used to build image stacks, reduce imaging noise, and generate 3D reconstructions^35^. A shell script was run to float images to a common density range, following which they were converted to a size 8 bit smoothed data MRC file as reported previously^17^. A 24″ Wacom tablet with interactive pen tool was used to construct 3D models from the MRC file open in IMOD. The image stack was scrolled through to locate a feature of interest e.g. the GBM. The pen tool was positioned over this feature and a contour drawn around the entire structure. The image stack was moved up or down by one section and a second contour was drawn around the same feature. This process was repeated on every image for the desired number of sections.

### Statistical analysis

170 measurements of podocyte FP density and 70 measurements of GBM thickness were taken per group. All identified invasions were quantified and invasion number was normalised to the total volume of glomerulus analysed. Comparisons between individual disease models and wild type were performed in GraphPad Prism using one way ANOVA with a Tukey’s post-test, *p* values less than 0.05 were considered to be significant.

## Additional Information

**How to cite this article**: Randles, M. J. *et al*. Three-dimensional electron microscopy reveals the evolution of glomerular barrier injury. *Sci. Rep.* **6**, 35068; doi: 10.1038/srep35068 (2016).

## Supplementary Material

Supplementary Information

Supplementary Movie S1

Supplementary Movie S2

Supplementary Movie S3

Supplementary Movie S4

Supplementary Movie S5

Supplementary Movie S6

Supplementary Movie S7

Supplementary Movie S8

Supplementary Movie S9

Supplementary Movie S10

Supplementary Movie S11
