# Supplementary material for: Three-dimensional electron microscopy reveals the evolution of glomerular barrier injury
Source: Sci Rep. 2016 Oct 11;6:35068. doi: 10.1038/srep35068 (PMC5057164; doi:10.1038/srep35068)
Supplement: Supplementary Information [file srep35068-s1.pdf]

Supplementary information

**Three-dimensional electron microscopy reveals the evolution of glomerular barrier injury**

Michael J Randles[1,2,+], Sophie Collinson[1,+], Tobias Starborg[1,3], Aleksandr Mironov[3], Mira Krendel[4], Eva Königshausen[5], Lorenz Sellin[5], Ian SD Roberts[6], Karl E Kadler[1], Jeffrey H Miner[7] and *Rachel Lennon[1,2,]

*[1]Wellcome Trust Centre for Cell-Matrix Research, Faculty of Life Sciences, University of Manchester, UK [2]Institute of Human Development, Faculty of Medical & Human Sciences, University of Manchester, UK [3]Electron Microscopy Core Facility, Faculty of Life Sciences, University of Manchester, UK [4]Department of Cell and Developmental Biology, SUNY Upstate Medical University, Syracuse, New York, USA [5]Department of Nephrology, Medical School, Heinrich Heine University, Düsseldorf, Germany [6]Department of Cellular Pathology, John Radcliffe Hospital, Oxford, UK [7]Division of Nephrology, Washington University School of Medicine, St Louis, Missouri, USA.*

+ these authors contributed equally to this work

*Corresponding author: Dr Rachel Lennon, Wellcome Trust Centre for Cell-Matrix Research Michael Smith Building, University of Manchester, Manchester M13 9PT, UK. Phone: 0044 (0) 161 2755498 Fax: 0044 (0) 161 2755082. Email: Rachel.Lennon@manchester.ac.uk

**Supplementary Movie S1:** Three-dimensional reconstruction of podocyte FPs from wild type C57BL/6JOlaHsd mice. Podocyte foot processes from two cells are shown (green and magenta respectively) and the glomerular basement membrane (blue). Scale bar represents 1 µm.

**Supplementary Movie S2:** Zoomed inset highlights focal podocyte foot process effacement, GBM thickening and loosening in young *Col4a3-/-* mice. Scale bar represents 1 µm.

**Supplementary Movie S3:** Zoomed inset highlights focal podocyte foot process effacement, GBM thickening and loosening in adult *Col4a3-/-* mice. Scale bar represents 1 µm.

**Supplementary Movie S4:** Zoomed inset highlights podocyte foot process invasion (indicated by arrow) in young *Col4a3-/-* mice. Scale bar represents 1 µm.

**Supplementary Movie S5:** Zoomed inset highlights podocyte foot process invasion (indicated by arrow) in adult *Col4a3-/-* mice. Scale bar represents 1 µm.

**Supplementary Movie S6:** Zoomed inset highlights podocyte foot process invasion in adult *Myo1e-/-* mice. Scale bar represents 1 µm.

**Supplementary Movie S7:** Zoomed inset highlights electron lucent regions of GBM in adult *Ptpro-/-* mice. Scale bar represents 1 µm.

**Supplementary Movie S8:** Zoomed inset highlights mesangial interposition within the GBM of wild type C57BL/6JOlaHsd mice. Scale bar represents 1 µm.

**Supplementary Movie S9:** Zoomed inset highlights mesangial invasion along the mesangial aspect of the GBM in adult *Col4a3-/-* mice. Scale bar represents 1 µm.

**Supplementary Movie S10:** Zoomed inset highlights mesangial invasion along the mesangial aspect of the GBM in adult *Ptpro-/-* mice. This mesangial cell invasion penetrates through the endothelial cell layer (arrow inset). Scale bar represents 1 µm.

**Supplementary Movie S11:** Zoomed inset highlights mesangial invasion along the mesangial aspect of the GBM in adult *Myo1e-/-* mice. Scale bar represents 1 µm.

## Supplemental figure S1

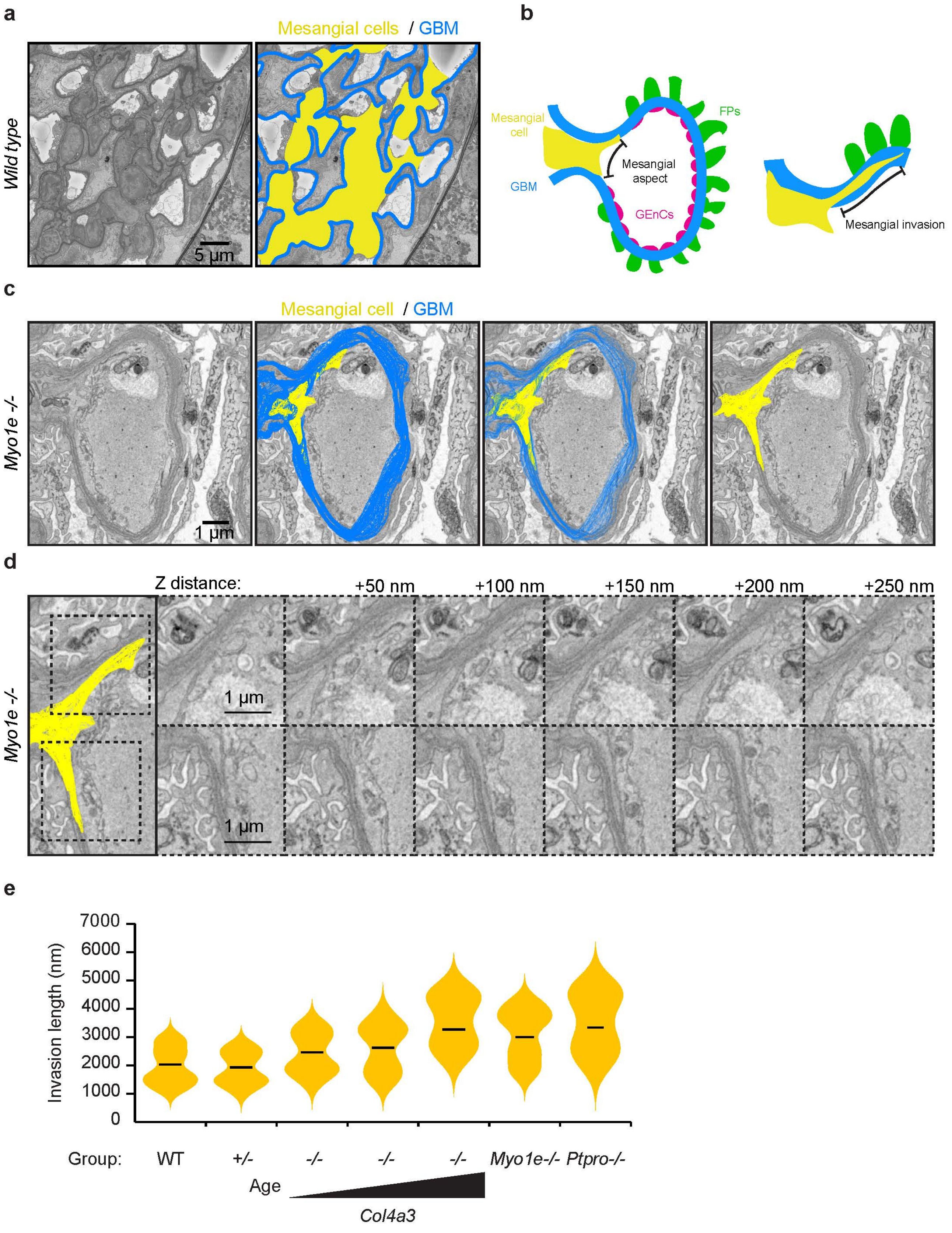


Mesangial cell invasions of the GBM. **(A)** Location of mesangial cells (yellow) relative to the GBM (blue) within the glomerulus. These cells and their associated ECM hold the capillary loops together. **(B)** Cartoon representation of the location of the mesangial cells (yellow), aspect (black line) and areas where mesangial invasion occur (black line). Relative positions of podocyte FPs (green), glomerular endothelial cells (magenta) and the GBM (blue) are shown. **(C)** Reconstructed SBF-SEM of mesangial invasion inside the GBM along the mesangial aspect in *Myo1e-/-* mice. Mesagial invasion is show in yellow and the GBM in blue. **(D)** Step through SBF-SEM showing the evolution of mesangial cell invasion (yellow) of the GBM along the mesangial aspect. **(E)** Quantification of the length of mesangial cell invasions. The violin plots describe the distributions for the length of mesangial cell invasion. The black lines represent the mean length of mesangial cell invasion of each group. GBM, glomerular basement membrane; FPs, foot processes; GEnCs, glomerular endothelial cells.

Supplemental figure S2

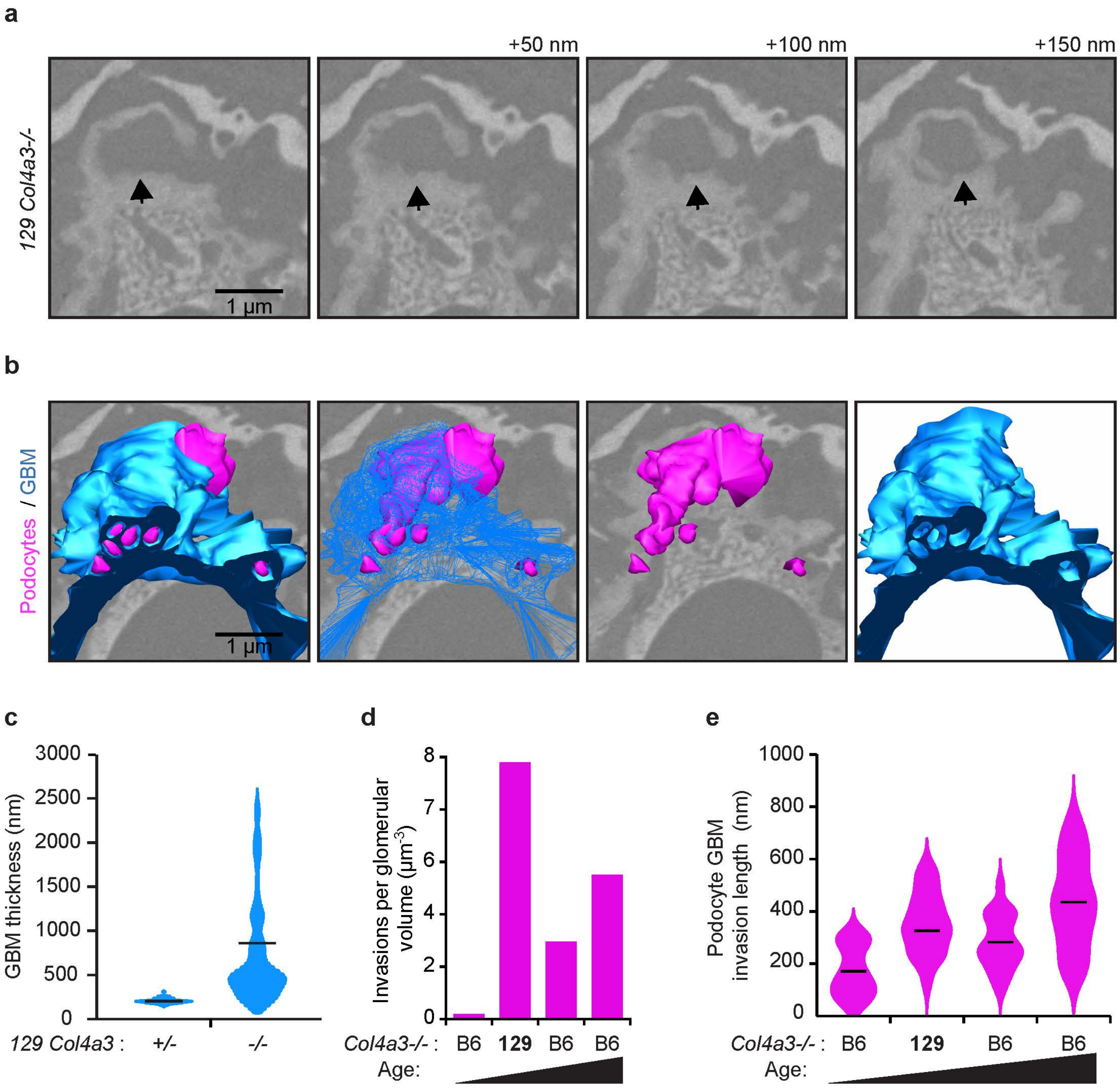


Podocytes invade the GBM in 129/Sv *Col4a3-/-* mice. **(A)** SBF-SEM showing podocyte invasion inside the GBM of 129/Sv *Col4a3-/-* mice. **(B)** Reconstructed SBF-SEM of podocyte invasion (magenta) inside the GBM (blue). **(C)** Quantification of GBM thickness. The violin plots describe the distributions for the thickness of GBM along the y-axis, the black lines represent the mean thickness of the GBM in each group. **(D)** 129/Sv mice have an accelerated accumulation of podocyte invasions at a younger age relative to B6 *Col4a3-/-* mice. **(E)** Quantification of podocyte FP invasions. The violin plots describe the distributions for the length of podocyte invasion into the GBM along the y-axis, the black lines represent the mean length of podocyte GBM invasions in each group. GBM, glomerular basement membrane; FPs, foot processes.

## Supplemental figure S3

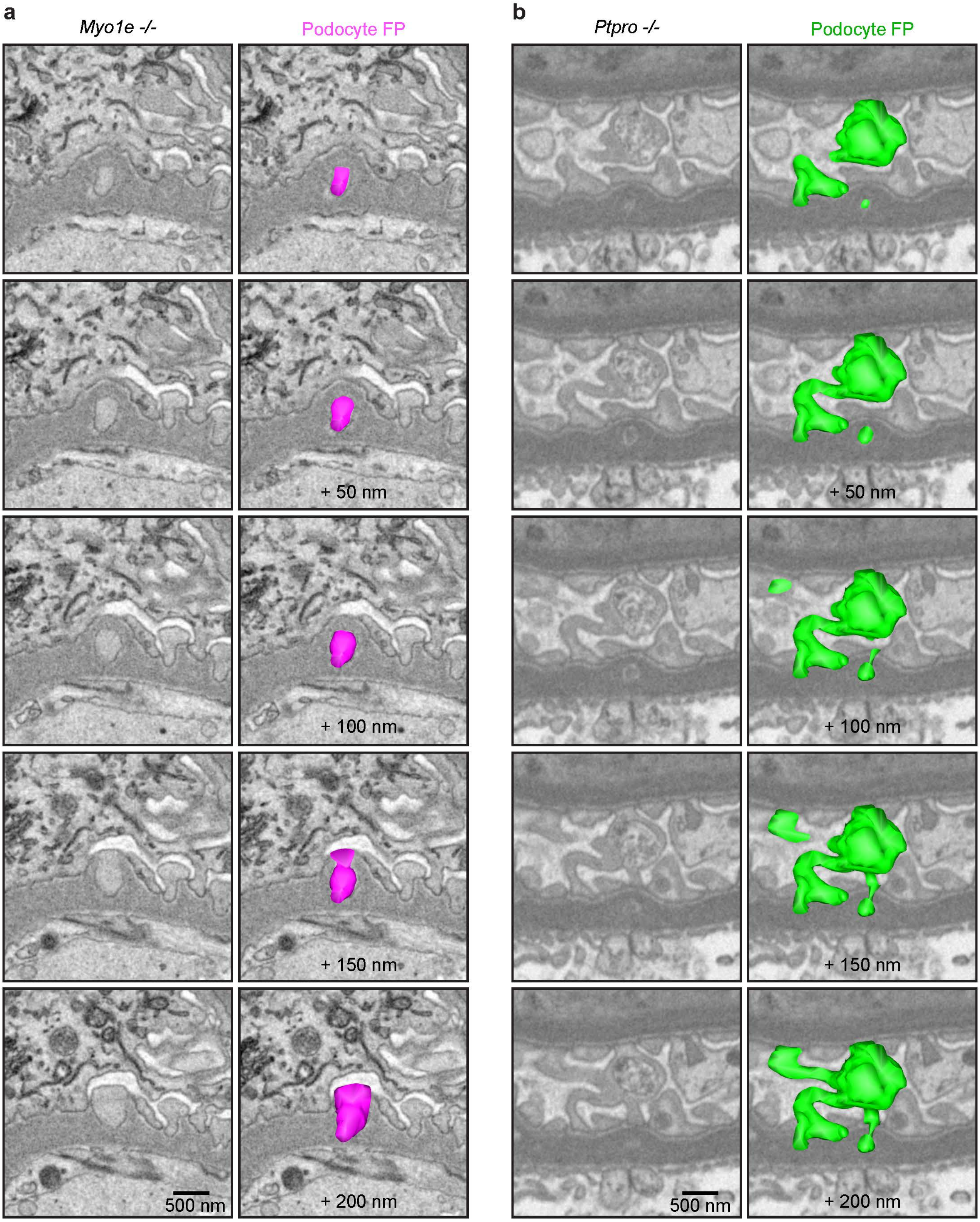


Tracking of cellular material embedded within the GBM through 50 nm sections with SBF-SEM reveals that cellular material within the capillary loop GBM originates from podocyte FPs in **(A)** *Myo1e-/-* and **(B)** *Ptpro-/-* mice. GBM, glomerular basement membrane; FPs, foot processes.

## Supplemental figure S4

**a**

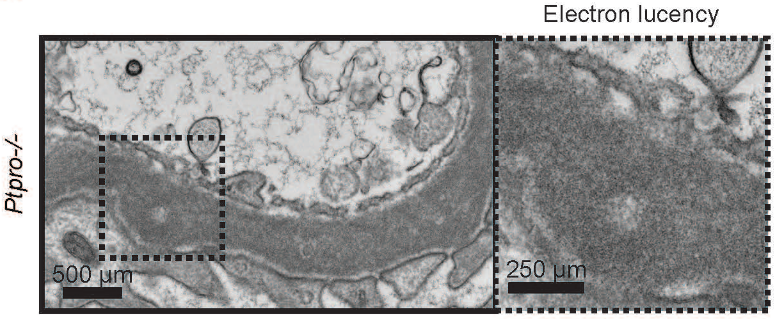


**b**

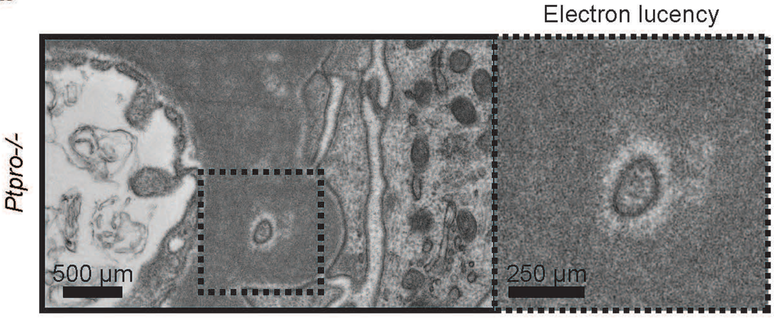


Transmission electron microscopy demonstrating electron lucent area within the GBM. (A) these occur in regions prior to **(A)** and surrounding **(B)** the site of podocyte invasions. GBM, glomerular basement membrane.
